# Efficacy and Safety of Electrosurgical Balloon-Assisted Leaflet Modification to Prevent Coronary Obstruction During Transcatheter Aortic Valve Replacement

**DOI:** 10.1016/j.shj.2025.100790

**Published:** 2025-12-26

**Authors:** Mostafa Naguib, Chantal Y. Asselin, Robert Kipperman, Leo Marcoff, Kostantinos P. Koulogiannis, Linda Gillam, Benjamin van Boxtel, John Brown, Philippe Généreux, Gennaro Giustino

**Affiliations:** Gagnon Cardiovascular Institute, Atlantic Health System, Morristown, New Jersey, USA

**Keywords:** Coronary obstruction, Electrosurgical traversal, Leaflet modification, Multidetector computed tomography, Procedural safety, UNICORN technique, Valve-in-valve, Transcatheter aortic valve replacement (TAVR)

## Abstract

**Background:**

Coronary obstruction (CO) during transcatheter aortic valve replacement (TAVR) is associated with significant morbidity and mortality. UNICORN (Undermining Iatrogenic Coronary Obstruction with Radiofrequency Needle) is a novel technique designed to prevent CO by performing electrosurgical leaflet traversal followed by intraleaflet valve implantation or complete leaflet laceration accomplished using noncompliant balloons. However, its efficacy and safety are not well established.

**Methods:**

We retrospectively reviewed all patients who underwent UNICORN-assisted TAVR for both valve-in-valve and native valve procedures at a single high-volume center between September 2024 and September 2025. Patients were selected based on preprocedural cardiac computed tomography demonstrating high anatomic risk for CO. In all cases, the target leaflet was traversed using an electrified 0.014″ wire, followed by serial noncompliant balloon dilatations of the leaflet to either achieve complete leaflet laceration or to accommodate for intra-leaflet valve implantation. Balloon-expandable valves were used in all procedures.

**Results:**

Fifteen patients underwent UNICORN-assisted TAVR. Twelve were valve-in-valve cases, and 3 involved native valves. The right coronary cusp was targeted in 11 procedures and the left in 6, including 2 requiring bileaflet modification. Technical success was achieved in all cases (100%). Procedural success was achieved in 93.3%. One patient developed acute CO due to skirt-related occlusion after a high implant in a degenerated self-expanding valve, requiring single-vessel coronary artery bypass surgery. No bailout coronary stenting was required. There were no in-hospital deaths or disabling strokes. All patients were alive at 30-day follow-up.

**Conclusions:**

In this single-center experience, UNICORN appears technically reproducible, effective in preventing CO, and safe in TAVR patients at high-risk for CO.

## Highlights

• UNICORN (Undermining Iatrogenic Coronary Obstruction with Radiofrequency Needle) technique is technically reproducible and effective in preventing coronary obstruction.
• UNICORN is feasible for valve-in-valve or native valve procedures.
• UNICORN is a complementary technique to Bioprosthetic or native Aortic Scallop Intentional Laceration to prevent Iatrogenic Coronary Artery obstruction for leaflet modification during transcatheter aortic valve replacement.

## Introduction

Iatrogenic coronary obstruction (CO) remains a rare but potentially devastating complication of transcatheter aortic valve replacement (TAVR), with an incidence of <1% in native valve cases and up to 3.5% in valve-in-valve (ViV) procedures.^1^, ^2^, ^3^ Preprocedural multidetector computed tomography (MDCT) enables reliable identification of patients at increased risk of CO.^1^, ^2^, ^3^, ^4^ The most frequently adopted strategies to prevent CO during TAVR are the chimney or snorkeling stenting^5^ and the Bioprosthetic or native Aortic Scallop Intentional Laceration to prevent Iatrogenic Coronary Artery obstruction (BASILICA).^6^ Chimney stenting consists of the implantation of a coronary stent in the coronary ostium protruding in the ascending aorta, maintaining proximal coronary patency. Conversely, BASILICA involves the electrosurgical traversal and laceration of the leaflet. Major limitations of snorkeling stenting include stent distortion, restenosis, thrombosis, and, at times, prohibitive coronary re-access. Limitations of BASILICA include its technical complexity, learning curve, and sometimes inadequate leaflet splitting necessitating bailout coronary stenting.^6^, ^7^, ^8^, ^9^

The UNICORN (Undermining Iatrogenic Coronary Obstruction with Radiofrequency Needle) technique represents a novel leaflet modification strategy that involves the electrosurgical traversal of the leaflet followed by serial balloon dilatations prior to valve deployment.^10^^,^^11^ With this technique, the same wire that is used for leaflet traversal is also used for valve implantation, reducing the need for additional arterial vascular access and streamlining the steps between leaflet traversal and valve implantation. This approach possibly offers a technically simpler and more reproducible alternative to BASILICA. However, data on the procedural efficacy and safety of UNICORN remain sparse. In the current manuscript, we present the largest single-center experience to date using the UNICORN technique in patients undergoing TAVR at high risk for CO, detailing technical feasibility, procedural outcomes, and early safety outcomes.

## Methods

**Figure 1:**
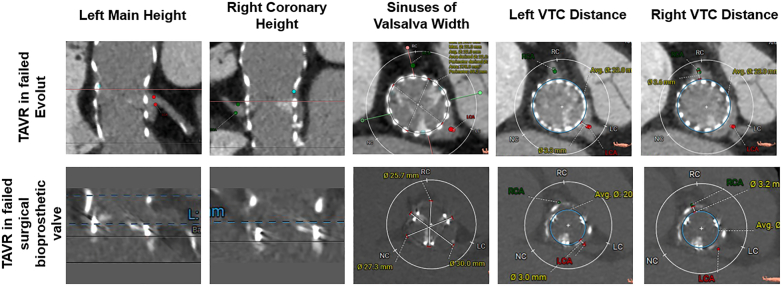
**Representative preprocedural MDCT. Top row (left to right):** procedural planning for TAVR-in-TAVR with MDCT images including left coronary artery height, right coronary artery height, SoV width, left VTC distance, and right VTC distance. **Bottom row (left to right):** procedural planning for TAVR-in-SAVR with MDCT images including left coronary artery height, right coronary artery height, SoV width, left VTC distance, and right VTC distance. Abbreviations: LC, left cusp; MDCT, multidetector computed tomography; NC, non-coronary cusp; RC, right cusp; SoV, sinus of Valsalva; TAVR, transcatheter aortic valve replacement; VTC, valve-to-coronary.

We retrospectively identified all patients who underwent UNICORN-assisted TAVR at our institution between September 2024 and September 2025. This cohort included both native and ViV procedures. Preprocedural MDCT was reviewed to assess high-risk factors for CO, including coronary ostial height <10 mm, virtual transcatheter valve-to-coronary distance < 4 mm, and leaflet length relative to coronary height, among others. Representative MDCT imaging are illustrated in Figure 1.

**Figure 2:**
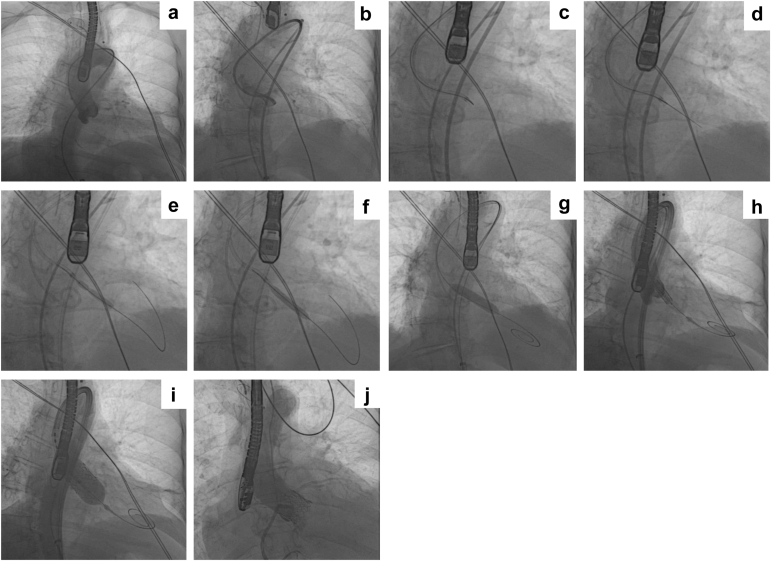
**Representative UNICORN-assisted TAVR procedure in a native aortic valve. (a)** Pigtail catheter placement and CEPD in appropriate position. **(b)** Visualization of the left coronary cusp (LCC). **(c)** Catheter aligned with LCC. **(d)** LCC traversal with Astato-Turnpike using electrosurgery at 50 W. **(e)** 8.0-mm non-compliant (NC) balloon inflation with a focal waist at LCC. **(f)** Fully inflated 8.0-mm NC balloon across the LCC. **(g)** Inflation of a 10.0 mm NC balloon. **(h)** Intraleaflet positioning of the SAPIEN 3 TAVR valve. **(i)** Intraleaflet deployment of the transcatheter heart valve. **(j)** Aortogram with the TAVR valve in excellent position with preserved coronary flow. Abbreviations: CEPD, cerebral embolic protection device; LCC, left coronary cusp; NC, non-compliant; TAVR, transcatheter aortic valve replacement; UNICORN, Undermining Iatrogenic Coronary Obstruction with Radiofrequency Needle.

**Figure 3:**
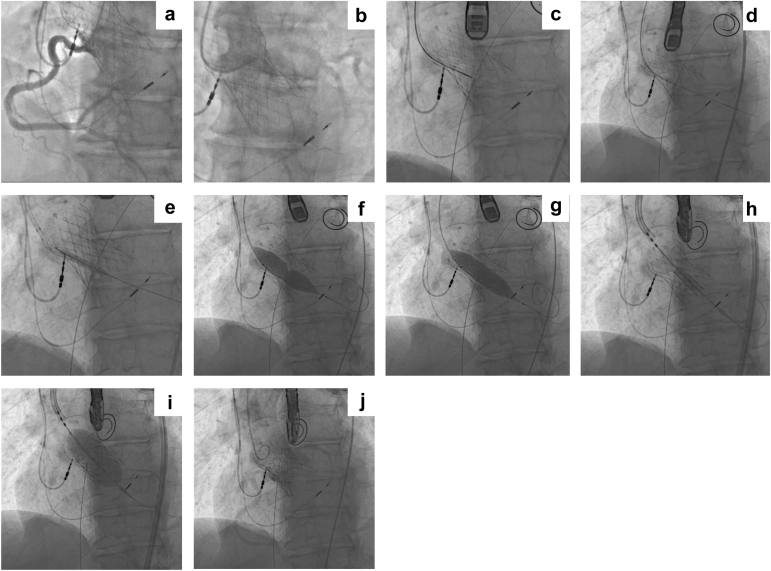
**Representative UNICORN-assisted TAVR procedure in a prior TAVR valve (TAVR-in-TAVR). (a)** Visualization of patent right coronary artery. **(b)** Visualization of patent left coronary artery. **(c)** Catheter aligned with right coronary cusp (RCC) with radiofrequency needle. **(d)** RCC traversal with Astato-Turnpike radiofrequency needle using electrosurgery at 50 W. **(e)** 5.0 mm non-compliant (NC) balloon inflation at RCC. **(f)** 16.0 mm NC balloon inflation with a focal waist at RCC. **(g)** RCC laceration with a fully inflated 16.0 mm NC balloon. **(h)** Positioning of 26 mm SAPIEN 3 Ultra RESILIA TAVR. **(i)** Deployment of the transcatheter heart valve. **(j)** Aortogram with TAVR-in-TAVR in good position, with preserved coronary flow. Abbreviations: NC, non-compliant; RCC, right coronary cusp; TAVR, transcatheter aortic valve replacement; UNICORN, Undermining Iatrogenic Coronary Obstruction with Radiofrequency Needle.

**Figure 4:**
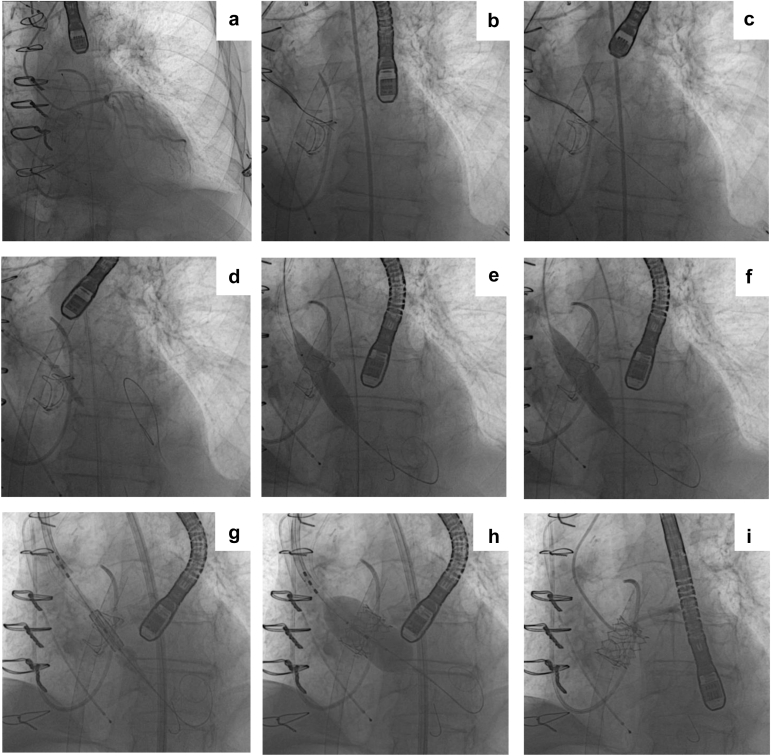
**Representative UNICORN-assisted TAVR procedure in a prior TAVR valve (TAVR-in-SAVR). (a)** Catheter placement within patent LM with visualized Magna Ease bioprosthetic valve. **(b)** Catheter aligned with the left coronary cusp (LCC). **(c)** LCC traversal with Astato-Turnpike using electrosurgery at 50 W. **(d)** Initial 5.0-mm non-compliant (NC) balloon inflation across the LCC. **(e)** 16.0-mm NC balloon inflation with a focal waist at LCC. **(f)** LCC laceration with fully inflated 16.0-mm NC balloon. **(g, h)** Deployment of the transcatheter heart valve. **(i)** Final angiogram demonstrating preserved coronary flow. Abbreviations: LCC, left coronary cusp; NC, non-compliant; TAVR, transcatheter aortic valve replacement; UNICORN, Undermining Iatrogenic Coronary Obstruction with Radiofrequency Needle.

**Figure 5:**
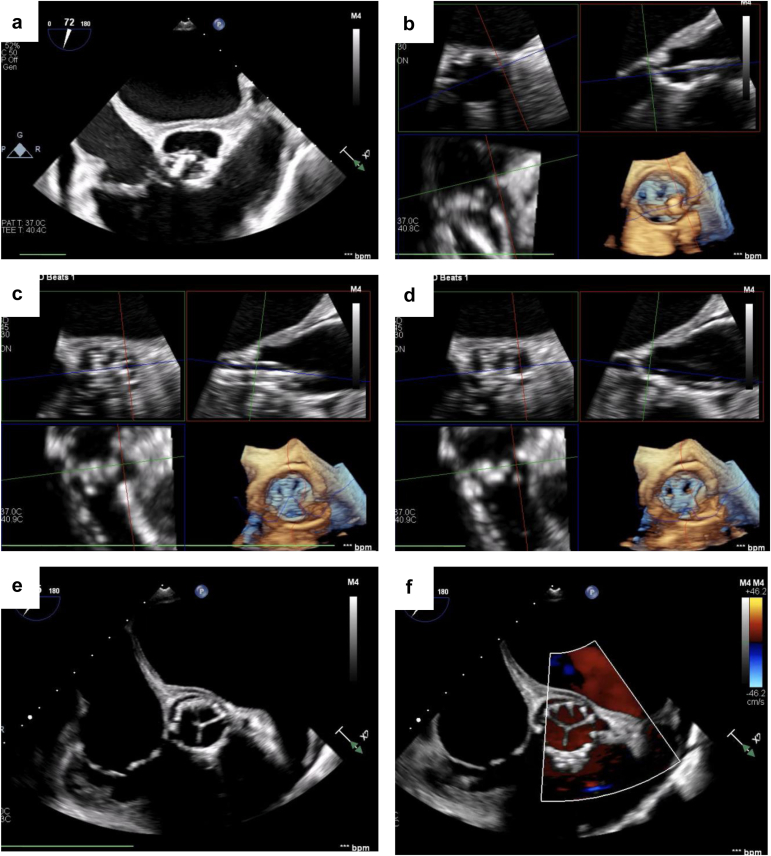
**Representative transesophageal echocardiographic imaging of UNICORN-assisted TAVR deployment in a native aortic valve. (a)** Mid-esophageal aortic valve short axis demonstrating calcified aortic valve. **(b)** Leaflet modification using the UNICORN technique. **(c)** Deployment of the transcatheter heart valve. **(d-f)** Final position of the implanted TAVR valve. Abbreviations: TAVR, transcatheter aortic valve replacement; UNICORN, Undermining Iatrogenic Coronary Obstruction with Radiofrequency Needle.

All cases were performed using a modified version of the original UNICORN technique, as recently reported by our group.^12^ In brief, the technique consists of 2 steps: electrosurgical leaflet traversal and balloon-assisted leaflet laceration. Leaflet traversal can be performed directly from the valve delivery sheath as the wire used for leaflet laceration will serve to deliver the transcatheter heart valve. The cusp plane should be first defined with 2 cusp injections through the guide-catheter in the side view and the front view. Then, a 0.014-inch Astato XS 20 guidewire (Asahi Intec USA, Inc., Irvine, California) is advanced within a microcatheter to the target leaflet hinge-point under angiographic guidance. The back-end of the Astato wire is then denuded using a scalpel blade which is then clamped to an electrosurgical pencil set with “cut” mode at 50-70 W. After achieving optimal positioning with fluoroscopy and transesophageal echocardiography guidance, the wire is electrified and advanced through the leaflet into the left ventricular outflow tract and left ventricle. Then, over the 0.014-inch wire a first dilatation of the leaflet can be performed using a 4.0 or 5.0 × 30 mm noncompliant balloon (Figure 2, Figure 3, Figure 4). Following the first balloon inflation, the 0.014-inch wire is exchanged with a stiff 0.035-inch wire (e.g. Supra Core guidewire) using a 0.035-inch–compatible microcatheter (e.g. Quick-Cross or Navicross catheter). Over the stiff 0.035-inch wire, a noncompliant 10- to 16-mm balloon is advanced across the leaflet hole created with the coronary balloon. This balloon will be used to either complete the leaflet laceration or to create space for the intraleaflet implantation of the transcatheter valve. Representative fluoroscopic images are illustrated in Figure 2 for native AV procedures (and Online Supplemental Videos 1-3). Representative echocardiographic images of UNICORN-assisted TAVR are illustrated in Figure 5 and Online Supplemental Video 4. Representative fluoroscopic images of valve-in-TAVR are illustrated in Figure 3 along with Online Supplemental Videos 5-7. Representative fluoroscopic images of valve-in-surgical bioprosthetic aortic valve are illustrated in Figure 4 along with Supplemental Videos 8-10.

### Data Collection

Baseline clinical characteristics were collected from electronic health records. Institutional review board oversight and waiver from consent was obtained per our institutional policies. Procedural characteristics collected included total fluoroscopic time (minutes), target cusp (right or left) for UNICORN, use of cerebral embolic protection devices, and individual anatomical characteristics. Several parameters of intraprocedural time intervals were recorded including traversal to laceration time (defined as the initial time the radiofrequency needle crosses the valve leaflet to balloon laceration of the leaflet), laceration to TAVR time (defined as the time from deflation of the last balloon laceration to deployment of the TAVR valve), and traversal to TAVR time (defined as the initial time the radiofrequency needle crosses the valve leaflet to deployment of the TAVR valve).

Technical success was defined as successful UNICORN traversal and balloon modification of the intended leaflet with successful access, delivery, and retrieval of the UNICORN system. Procedural success, measured at the exit from the hybrid operative room, was defined as successful UNICORN traversal and balloon modification of the intended leaflet, successful access, delivery, and retrieval of the UNICORN device system, successful TAVR device implantation, absence of procedural mortality, absence of coronary artery obstruction, and freedom from emergency cardiac surgery or reintervention related to the UNICORN technique. Major adverse cardiovascular events (MACE) was defined as a composite of cardiovascular death, nonfatal myocardial infarction (MI), and nonfatal stroke. CO was considered a component of MACE if it resulted in MI, cardiovascular death, or required emergent coronary intervention.

### Statistical Analysis

Continuous variables were summarized as means ± standard deviations or medians with interquartile ranges, and categorical variables as counts and percentages. The occurrence of MACE was reported descriptively. Given the sample size and absence of a comparator group, no formal hypothesis testing was performed.

## Results

A total of 15 patients who underwent UNICORN-assisted TAVR for both ViV and native procedures between September 2024 and September 2025 at a single tertiary structural heart disease center were identified and included in the study. Baseline characteristics are summarized in Table 1. The mean age of the patients was 81.7 years, the majority were female (66.7%), with body mass index of 27.1 kg/m^2^. All patients were in New York Heart Association functional class III or IV. Aortic stenosis (AS) accounted for the primary AV dysfunction in 66.7% of patients, while 26.7% were attributed to severe aortic regurgitation (AR); the remaining 6.7% had mixed disease with both severe AS and severe AR. Baseline echocardiographic data is reported in Table 1. All patients were deemed at prohibitive surgical risk with a median Society of Thoracic Surgeons score of 12%. The MDCT imaging characteristics are summarized in Table 2. The median aortic annulus area was 366.5 mm^2^. In patients undergoing UNICORN, the median left coronary height was 10.2 and 9.0 mm on the right. For ViV procedures, the median left-sided virtual transcatheter valve-to-coronary distance was 3.8 mm, and 2.5 mm on the right. Individual MDCT characteristics and the predicted mechanism of CO in each patient are reported in Table 3.

**Table 1:** Baseline characteristics

Characteristics	N = 15
Age, yrs	81.7 ± 8.1
Female, n (%)	10 (66.7)
BMI, kg/m^2^	27.2 ± 4.8
STS score, %	12 [7.9 – 22.4]
NYHA functional class III or IV, n (%)	15 (100)
Coronary and revascularization history	
Prior PCI, n	2
Prior coronary ostial stenting, n	0
Prior CABG, n	0
Documented CTO, n	2
TAVR setting, n (%)	
Native	3 (20)
Valve in TAVR	9 (60)
Valve in SAVR	3 (20)
Valve type, n (%)	
CoreValve/Evolut	4 (26.7)
Evolut Pro Plus	4 (26.7)
Sapien 3	1 (6.7)
Magna Ease	3 (20)
Valve nominal size, n (%)	
20 mm	2 (13.3)
23 mm	1 (6.7)
25 mm	1 (6.7)
26 mm	3 (20)
29 mm	3 (20)
34 mm	2 (13.3)
Aortic valve dysfunction, n (%)	
Aortic stenosis	10 (66.7)
Aortic regurgitation	4 (26.7)
Mixed	1 (6.7)
AV peak velocity, m/s	4.0 [3.7 – 4.4]
AV mean gradient, mmHg	37.0 [31.0 – 46.0]
AVA, cm^2^	0.8 [0.6 – 1.2]

Continuous variables are reported as mean ± SD if normally distributed, or as median [interquartile range] if not normally distributed. Categorical variables are reported as n (%).

Abbreviations: AV, aortic valve; AVA, aortic valve area; BMI, body mass index (calculated as weight in kilograms divided by the square of height in meters); CABG, coronary artery bypass grafting; CTO, chronic total occlusion; NYHA, New York Heart Association; PCI, percutaneous coronary intervention; STS, Society or Thoracic Surgeons; TAVR, transcatheter aortic valve replacement.

**Table 2:** Coronary obstruction risk assessed by preprocedural MDCT

	N = 15
Coronary artery height, mm	
Left	10.2 [5.6 – 11.6]
Right	9.0 [6.1 – 14.6]
VT-STJ, mm	
Left	0 [0 – 0.9]
Right	0 [0 – 0.8]
STJ height, mm	
Left	19.7 [17.5 – 23.0]
Right	19.7 [16.0 – 23.0]
SoV width, mm	
Left	26.1 [25.1 – 30.2]
Right	25.7 [25.0 – 28.9]
VTC distance, mm	
Left	3.8 [2.7 – 4.7]
Right	2.5 [1.3 – 3.5]
Aortic annulus area, mm^2^	366.5 [342.8 – 393.0]

Continuous variables are reported as median [interquartile range] if not normally distributed.

Abbreviations: MDCT, multidetector computed tomography; SoV, sinus of Valsalva; STJ, sinotubular junction; VTC, valve-to-coronary; VT-STJ, virtual transcatheter heart valve-to-sinotubular junction.

**Table 3:** Individualized CT baseline characteristics

Patient ID	Aortic annulus area (mm^2^)	Coronary artery height (mm)		VTSTJ (mm)		VTC (mm)		SoV width (mm)		Predicted mechanism of obstruction
		Left	Right	Left	Right	Left	Right	Left	Right	
1	343.5	1.3	6.1	1.4	0.9	3.7	5.0	23.0	25.0	Direct
2	360.4	5.2	5.6	0	0	3.3	3.6	26.0	27.0	Sinus sequestration
3	361	4.8	7.0	0	0	3.0	3.2	30.0	25.7	Sinus sequestration
4	379	9.4	12	--	--	--	--	26.1	23.2	Mixed
5	347.9	16.3	5.5	0.9	1.1	5.5	0	--	--	Direct
6	367	10.2	15.7	--	--	--	--	24.6	25.4	Mixed
7	440.6	5.6	8.0	2	2.5	2	3.3	--	--	Direct
8	522.6	6.2	9.0	0	0.5	2.6	0	32.5	30.2	Sinus sequestration
9	340.6	11.6	9.2	--	--	--	--	25.5	25.0	Mixed
10	365.9	16.4	13.1	0	0	2.0	2.1	--	--	Mixed
11	323	10.7	18.8	0	0	3.9	2.9	28.6	27.6	Sinus sequestration
12	426	11.0	15.0	0	0	7.2	5.1	--	--	Sinus sequestration
13	378	17.9	14.6	0.9	0	4.9	1.1	--	--	Sinus sequestration
14	336	10.9	6.2	0.5	0	4.2	1.9	--	--	Sinus sequestration
15	373	9.0	5.0	0	0	4.2	1.7	30.4	30.2	Mixed

Normally distributed continuous variables are reported at mean ± SD, or as median ± interquartile range if not normally distributed. Categorical variables are reported as n (%).

Abbreviations: CT, computed tomography; SoV, sinus of Valsalva; VTC, valve to coronary; VT-STJ, virtual transcatheter heart valve-to-sinotubular junction.

-- = not recorded.

Of the 15 patients who underwent UNICORN-assisted TAVR, 12 were ViV procedures (9 TAVR; 3 SAVR), while the remaining 3 patients underwent native-valve UNICORN. Among the 15 procedures, the right coronary cusp was the target leaflet in 11 cases and the left coronary cusp in 6; notably, 2 of these were double-UNICORN procedures in which both leaflets were modified. Leaflet traversal was successful in all cases. Procedural characteristics are summarized in Tables 4 and 5. The median fluoroscopic time was 40 minutes, and the median contrast volume was 175 mL. The median intraprocedural time of leaflet traversal to laceration was 13 minutes. The median laceration to TAVR time was 4 minutes, and the median traversal to TAVR deployment was 18 minutes. Both leaflet traversal and leaflet laceration were achieved with a median of 1 attempt per procedure. Preemptive mechanical circulatory support with left atrial venoarterial membrane oxygenation was required in 5 patients, prompted by either preexisting hemodynamic instability and/or anticipated hemodynamic compromise associated with leaflet modification. Procedural success was achieved in 93.3% of cases. One patient experienced acute CO due to high TAVR implant in a degenerated CoreValve resulting in direct occlusion of the coronary ostia by the skirt of the balloon-expandable valve, requiring emergent single-vessel bypass surgery (Supplemental Figure 1 and Supplemental Videos 11-14). There were no cases of bailout stenting. There were no in-hospital deaths or strokes. Three patients experienced a major bleed (one was non-procedure related from the gastro-intestinal tract and one was related to coronary artery bypass surgery). Cerebral protection was utilized in 53.3% of cases. All patients were successfully discharged without any further complications. There were no MACE or deaths at 30-day follow-up.

**Table 4:** Procedural characteristics

	N = 15
Valve type, n (%)	
SAPIEN 3 Resilia	15 (100%)
Valve nominal size, n (%)	
20 mm	7 (46.7)
23 mm	6 (40)
26 mm	2 (13.3)
29 mm	0 (0)
Access for TAVR, n (%)	
Transfemoral	15 (100)
Percutaneous axillary	0 (0)
Target cusp, n (%)	
Left	6 (40)
Right	11 (73.3)
Sentinel cerebral protection, n (%)	8 (53.3)
Mechanical circulatory support, n (%)	
IABP	0 (0)
Percutaneous LVAD (Impella CP)	0 (0)
LAVA-ECMO	5 (33.3)
Type of anesthesia, n (%)	
General anesthesia	15 (100)
Moderate sedation	0 (0)
Total fluoroscopic time, min	40 [24 – 60]
Time from leaflet traversal to laceration, min	13 [8 – 20]
Time from leaflet laceration to TAVR, min	4 [3 – 5]
Time from leaflet traversal to TAVR, min	18 [13 – 24]
Number of leaflet attempts, n	1 [1 – 2]
Total contrast volume, mL	175 [109 – 245]
Technical success∗, %	15 (100)
Coronary obstruction, %	1 (6.7)
Bailout stenting, %	0 (0)
Conversion to emergent surgical intervention, %	1 (6.7)
Procedural success†, %	14 (93.3)
30-d survival, %	15 (100)

Normally distributed continuous variables are reported as mean ± SD, or as median [interquartile range] if not normally distributed. Categorical variables are reported as n (%).

Abbreviations: IABP, intraaortic balloon pump; LAVA-ECMO, left atrial venoarterial extracorporeal membrane oxygenation; LVAD, left ventricular assist device; TAVR, transcatheter aortic valve replacement; UNICORN, Undermining Iatrogenic Coronary Obstruction with Radiofrequency Needle.

∗ Technical success was defined as: successful UNICORN traversal and balloon modification of the intended leaflet with successful access, delivery, and retrieval of the UNICORN device system.

† Procedural success was assessed upon exit from the hybrid operating room or cardiac catheterization laboratory and was defined as: successful access, delivery, and retrieval of the UNICORN device system, successful TAVR device implantation, absence of procedural mortality, absence of coronary artery obstruction, and freedom from emergency cardiac surgery or reintervention related to UNICORN-assisted TAVR procedure.

**Table 5:** Individual procedural characteristics and parameters

Patient ID	Traversal to laceration, (min)	Laceration to TAVR, (min)	Traversal to TAVR, (min)	Number of traversal attempts	Fluro time (min)	Contrast volume (mL)	CEPD	Technical success
1	25	3	28	5	40	80	Y	Y
2	24	2	26	7	80	250	Y	Y
3	10	2	12	2	38	100	Y	Y
4	58	5	63	1	60	245	Y	Y
5	2	3	5	1	84	180	N	Y
6	16	3	19	2	24	130	N	Y
7	8	6	14	1	25	109	Y	Y
8	20	4	24	11∗	60	400	Y	Y
9	8	5	13	1	24	80	Y	Y
10	8	5	13	1	61	340	N	Y
11	14	4	18	1	18.5	120	N	Y
12	13	2	15	1	23	120	N	Y
13	12	4	16	1	36	176	N	Y
14†	18	5	23	1	58	181	N	Y
15†	13	6	19	1	57	175	Y	Y

Continuous variables are reported as mean ± SD if normally distributed or as median [interquartile range] if not normally distributed. Categorical variables are reported as n (%).

Technical success was defined as: successful UNICORN traversal and balloon modification of the intended leaflet with successful access, delivery, and retrieval of the UNICORN device system.

Abbreviations: CEPD, cerebral embolic protection device; Fluro time, fluoroscopic time; N, No; TAVR, transcatheter aortic valve replacement; UNICORN, Undermining Iatrogenic Coronary Obstruction with Radiofrequency Needle; Y, Yes.

∗ In one patient, an increased number of leaflet traversal attempts was required due to a right-sided aortic arch and difficulty achieving leaflet traversal in an heavily calcified leaflet.

† Leaflet modification was performed on both the left and right coronary cusps. Reported time intervals and traversal attempts represent the mean values across both target leaflets.

## Discussion

CO remains one of the most devastating complications of TAVR, associated with high procedural mortality.^1^, ^2^, ^3^, ^4^ The expanding application of TAVR across native AVs and degenerative bioprosthetic valves has led to a growing population at risk for this complication.^1^, ^2^, ^3^, ^4^ This increasing risk underscores the importance of accurate preprocedural risk stratification and preventive strategies in contemporary TAVR practice.^4^^,^^13^

Building on the need for refined preventive approaches, we evaluated a modified UNICORN technique in patients undergoing TAVR at high risk of CO. Previously, a range of preventive strategies have been developed to mitigate CO risk in anatomically complex TAVR cases. Coronary protection with chimney or snorkel stenting remains a widely used option due to its procedural familiarity, but it does not address the underlying mechanism of obstruction—leaflet displacement—and may complicate future coronary access or affect long-term stent durability.^13^ In contrast, leaflet modification techniques aim to directly prevent obstruction by lacerating the leaflet prior to valve implantation. Among these, BASILICA employs electrosurgical leaflet laceration and has demonstrated efficacy in native AS and ViV TAVR.^6^, ^7^, ^8^, ^9^ However, procedural complexity, a substantial learning curve, and anatomic challenges—including commissural fusion, asymmetric leaflet orientation, and severely calcified or highly mobile leaflets—continue to limit broader adoption.^6^, ^7^, ^8^, ^9^^,^^14^^,^^15^ The ShortCut device offers a simplified approach for leaflet splitting and has shown promise in ViV TAVR,^14^ but published experience remains largely confined to ViV cases, with no validated use in native AS, and CO events have still been reported due to incomplete leaflet splitting and residual tissue impingement.^15^ Within this evolving landscape, we sought to evaluate a modified UNICORN technique as an alternative leaflet modification approach, consisting of electrosurgical leaflet traversal followed by balloon-assisted leaflet modification with potential applicability across a broad range of anatomies and substrates.

This initial single-center experience supports the feasibility and safety of this modified UNICORN technique as a viable alternative to BASILICA in selected TAVR patients at high-risk for CO. The high rate of technical success and absence of periprocedural mortality or stroke are encouraging, particularly given the anatomical complexity typically associated with elevated CO risk.^1^^,^^2^ Notably, the technique was effective in preventing CO and was associated with relatively short procedural times despite the early experience.

Compared to BASILICA, UNICORN overall offers several potential advantages: 1) leaflet modification can be performed directly from the valve delivery access, obviating the need for additional arterial access and possibly reducing vascular complications; 2) the wire utilized to perform UNICORN can also be maintained for valve delivery and implantation, thereby preventing loss of wire access to the left ventricle and shortening the time between leaflet laceration and valve implantation; 3) UNICORN obviates the need for snaring the traversal wire, creating a flying-V and performing a laceration in a flying-V configuration, thereby reducing procedural time and overall tool utilization. The pitfall of UNICORN is the less predictable leaflet laceration. In addition, this technique is ideal for patients with a single coronary at risk. If both coronaries are at risk, it should be combined with BASILICA or snorkeling stenting for the contralateral leaflet in order not to cause excessive hemodynamic instability from wide-open iatrogenic AR. In our series 2 patients underwent bilateral UNICORN. Both cases were failed self-expanding valve with high-risk of obstruction bilaterally. Both patients were placed on preemptive left atrial venoarterial membrane oxygenation in anticipation of hemodynamic instability created by bilateral UNICORN.

Leaflet modification with ShortCut has been shown to be effective and safe in producing a base-to-tip mechanical leaflet laceration and is technically easier and more reproducible than BASILICA. However, the main limitations of ShortCut remain its associated cost, the inability to effectively modify heavily calcified leaflets and potential interactions with valves with large frames (e.g. failed Evolut valves). In addition, similar to BASILICA, a linear laceration of the leaflet may remain ineffective at preventing CO in cases at high risk for occlusion.

In this early experience, we adopted a modified version of the original UNICORN technique recently described by our group.^12^ It is important to note that compared to the initial case report by Chan et al., our technique employs an electrified 0.014″ Astato wire for initial leaflet traversal, rather than the 0.035″ wire, similar to the first step of BASILICA. In our opinion, traversing with a 0.014” wire enables a safer and more precise leaflet traversal and potentially reduced risk of iatrogenic tissue injury resulting in major complications. The original UNICORN technique contemplated intraleaflet deployment of the transcatheter heart valve. Conversely, in our modified version, complete leaflet laceration was accomplished prior to valve implantation in all ViV cases using a large (14 or 16 mm) valvuloplasty balloon. In the 3 native TAVR cases, intraleaflet deployment was favored due to concerns for annular injury using a large valvuloplasty balloon potentially creating an irregular tear propagating toward the annulus rather than the free edge of the leaflet. In this series, a cerebral embolic protection device was employed whenever technically feasible. However, in select cases, sentinel delivery was not possible due to unfavorable aortic arch anatomy or subclavian/innominate vessel tortuosity, precluding safe device advancement.

To our knowledge, this report represents the largest single-center experience of patients undergoing UNICORN-assisted TAVR to date. As such, it provides meaningful early insight into the reproducibility and safety profile of this emerging technique in anatomically high-risk populations. Similar findings have been reported in an early-experience study from the Cleveland Clinic including 10 patients undergoing ViV TAVR.^16^ Future comparative studies with larger cohorts and dedicated bench testing are needed to further define optimal anatomic selection criteria and to rigorously compare UNICORN with other leaflet modification strategies.

## Study Limitations

This study has several limitations. First, it represents a retrospective analysis from a single, high-volume center, which may limit generalizability to other practice environments. The sample size remains modest and precludes definitive conclusions regarding rare adverse events or comparative efficacy versus other techniques. Additionally, follow-up was limited to the in-hospital period and 30-day outcomes; longer-term durability of leaflet modification and freedom from delayed coronary events remain unknown. Finally, procedural success may have been influenced by operator experience and institutional expertise with advanced TAVR planning and execution.

## Conclusion

In this early single-center experience, UNICORN-assisted TAVR was associated with high technical success and favorable short-term outcomes in patients at elevated risk for CO. This technique may serve as a simpler and more accessible alternative to BASILICA in select cases. Further studies are warranted to validate these findings in larger, multicenter cohorts and to evaluate its comparative effectiveness versus other leaflet modification strategies.

## Ethics Statement

Institutional review board oversight and waiver from consent was obtained per our institutional policies.

## CRediT Authorship Contributions

All authors made substantial contributions to the conception and design of the study, acquisition of data, analysis and interpretation of data, and drafting and editing the manuscript. All authors have read and agreed to the final version of the manuscript.

## Funding

The authors have no funding to report.

## Disclosure Statement

Kostantinos P. Koulogiannis served as a consultant for 10.13039/100006520Edwards Lifesciences, 10.13039/100004374Medtronic, and 10.13039/100000046Abbott; received equity from 4C medical. Linda Gillam received consulting or advisory fees from 10.13039/100004374Medtronic, Egnite, and 10.13039/100006775GE Healthcare; received funding grants from 10.13039/100004374Medtronic and 10.13039/100006520Edwards; received equity from 4C medical. Philippe Généreux received institutional research grants from 10.13039/100006520Edwards Lifesciences; received consulting fees from 10.13039/100011949Abbott Vascular, 10.13039/100020297AbioMed, 10.13039/100008497Boston Scientific Corporation, 10.13039/100016476Cardiovascular Systems, 10.13039/100006479Cordis, 10.13039/100006775GE Healthcare, iRhythm Technologies Inc, 10.13039/100006520Edwards Lifesciences, Egnite, Haemonetics, Shockwave, 10.13039/100004340Siemens, Soundbite Medical Inc, 10.13039/100004374Medtronic, Opsens, Puzzle Medical, Pi-Cardia, 4C medical, Saranas, and Teleflex; received equity from Soundbite Medical Inc, Saranas, Shockwave, Puzzle Medical, Pi-Cardia; served as a PI for Eclipse Trial, ALTA Valve Feasibility Study, and EARLY TAVR trial and PROGRESS trial, both sponsored by 10.13039/100006520Edwards Lifesciences; served as a proctor for 10.13039/100006520Edwards Lifesciences. Gennaro Giustino served as a proctor and consultant for 10.13039/100006520Edwards Lifesciences, 10.13039/100004374Medtronic, and Shockwave Medical; was a shareholder of Antegrade Medical.

The other authors had no conflicts to declare.

## Supplementary Data

Supplemental Figure 1 **Failed UNICORN-assisted TAVR-in-TAVR with post-TAVR coronary obstruction. (a)** Nonselective angiogram demonstrating patent right coronary artery. **(b)** Selective angiogram demonstrating patent left coronary artery. **(c)** left main protection with GuideLiner catheter and coronary stent (not deployed). **(d)** Catheter aligned with right coronary cusp. **(e)** right coronary cusp traversal with Astato-Turnpike using electrosurgery at 50 W. **(f)** Initial 5.0-mm balloon inflation with laceration of the right coronary cusp. **(g, h)** right coronary cusp laceration with 16.0-mm balloon. **(i, j)** Deployment of the transcatheter heart valve. **(k)** Aortogram with TAVR-in-TAVR and absent right coronary flow. **(l)** Selective angiogram with TAVR-in-TAVR and absent right coronary flow indicating right coronary obstruction. Abbreviations: TAVR, transcatheter aortic valve replacement; UNICORN, Undermining Iatrogenic Coronary Obstruction with Radiofrequency Needle.

Supplemental Video 1 Fluoroscopic imaging of UNICORN leaflet traversal in a native aortic valve. Fluoroscopic demonstration of electrosurgical traversal of the target aortic valve leaflet in a native aortic valve, establishing a stable rail for subsequent leaflet modification. Abbreviation: UNICORN, Undermining Iatrogenic Coronary Obstruction with Radiofrequency Needle.

Supplemental Video 2 Fluoroscopic imaging of UNICORN leaflet laceration in a native aortic valve. Fluoroscopic demonstration of intraleaflet balloon inflation to allow for intraleaflet deployment of the transcatheter heart valve. Abbreviation: UNICORN, Undermining Iatrogenic Coronary Obstruction with Radiofrequency Needle.

Supplemental Video 3 Fluoroscopic imaging of UNICORN-assisted intraleaflet THV deployment in a native aortic valve. Fluoroscopic sequence of UNICORN-assisted intraleaflet THV deployment in a native valve. Abbreviation: UNICORN, Undermining Iatrogenic Coronary Obstruction with Radiofrequency Needle.

Supplemental Video 4 UNICORN-assisted THV deployment in a native aortic valve on transesophageal echocardiogram. Transesophageal echocardiographic imaging of UNICORN-assisted THV deployment in a native aortic valve. Abbreviation: UNICORN, Undermining Iatrogenic Coronary Obstruction with Radiofrequency Needle.

Supplemental Video 5 Fluoroscopic imaging of UNICORN leaflet traversal in a TAVR-in-TAVR. Fluoroscopic demonstration of electrosurgical traversal of the target leaflet. Abbreviations: TAVR, transcatheter aortic valve replacement; UNICORN, Undermining Iatrogenic Coronary Obstruction with Radiofrequency Needle.

Supplemental Video 6 Fluoroscopic imaging of UNICORN leaflet laceration in a TAVR-in-TAVR. Fluoroscopic demonstration of leaflet laceration using a valvuloplasty balloon. Abbreviations: TAVR, transcatheter aortic valve replacement; UNICORN, Undermining Iatrogenic Coronary Obstruction with Radiofrequency Needle.

Supplemental Video 7 Fluoroscopic imaging of UNICORN-assisted TAVR-in-TAVR. Fluoroscopic sequence of TAVR-in-TAVR deployment following UNICORN-assisted leaflet modification. Abbreviations: TAVR, transcatheter aortic valve replacement; UNICORN, Undermining Iatrogenic Coronary Obstruction with Radiofrequency Needle.

Supplemental Video 8 Fluoroscopic imaging of UNICORN leaflet traversal in a TAVR-in-SAVR. Fluoroscopic demonstration of electrosurgical traversal of the target leaflet in a TAVR-in-SAVR. Abbreviations: TAVR, transcatheter aortic valve replacement; UNICORN, Undermining Iatrogenic Coronary Obstruction with Radiofrequency Needle.

Supplemental Video 9 Fluoroscopic imaging of UNICORN leaflet laceration in a TAVR-in-SAVR. Fluoroscopic demonstration of laceration of the target leaflet in a TAVR-in-SAVR. Abbreviations: TAVR, transcatheter aortic valve replacement; UNICORN, Undermining Iatrogenic Coronary Obstruction with Radiofrequency Needle.

Supplemental Video 10 Fluoroscopic imaging of UNICORN-assisted TAVR-in-SAVR. Fluoroscopic sequence of TAVR-in-SAVR deployment following UNICORN-assisted leaflet modification. Abbreviations: TAVR, transcatheter aortic valve replacement; UNICORN, Undermining Iatrogenic Coronary Obstruction with Radiofrequency Needle.

Supplemental Video 11 Baseline aortography with pigtail positioning in prior Evolut Pro TAVR. Aortography demonstrating pigtail position within the prior Evolut Pro with patent coronary flow. Abbreviation: TAVR, transcatheter aortic valve replacement.

Supplemental Video 12 Balloon-assisted laceration of the right coronary cusp leaflet. Mechanical RCC leaflet laceration is completed using a 16.0-mm balloon, achieving a full-thickness split along the traversal plane.

Supplemental Video 13 Transcatheter valve deployment following UNICORN leaflet modification. Deployment of a transcatheter heart valve within the previously implanted TAVR valve. Abbreviations: TAVR, transcatheter aortic valve replacement; UNICORN, Undermining Iatrogenic Coronary Obstruction with Radiofrequency Needle.

Supplemental Video 14 Selective coronary angiography confirming acute right coronary obstruction. Selective right coronary angiography confirming complete absence of RCA flow, consistent with hemodynamically significant right coronary obstruction following valve deployment.
