# Supplementary figures and images for: Efficacy and Safety of Electrosurgical Balloon-Assisted Leaflet Modification to Prevent Coronary Obstruction During Transcatheter Aortic Valve Replacement
Source: Struct Heart. 2025 Dec 26;10(3):100790. doi: 10.1016/j.shj.2025.100790 (PMC12878699; doi:10.1016/j.shj.2025.100790)

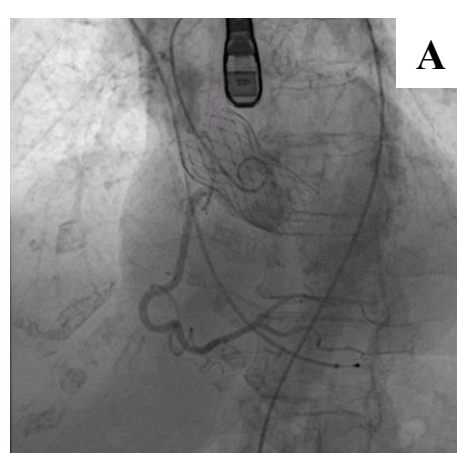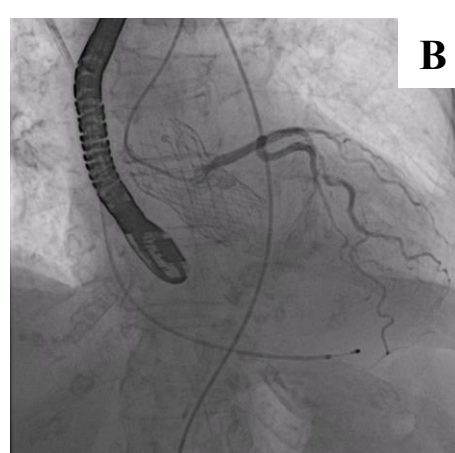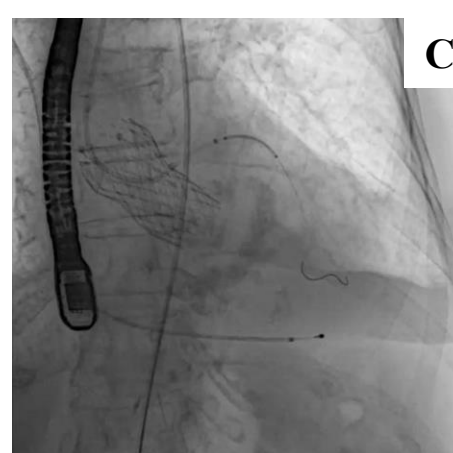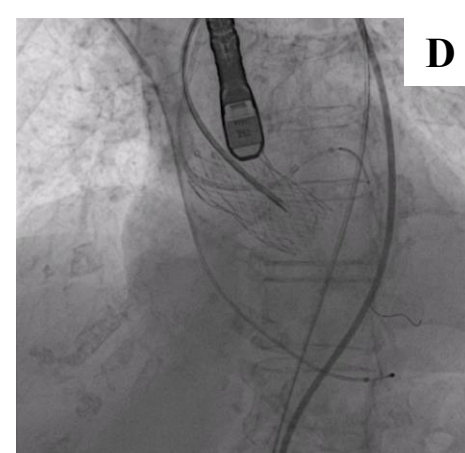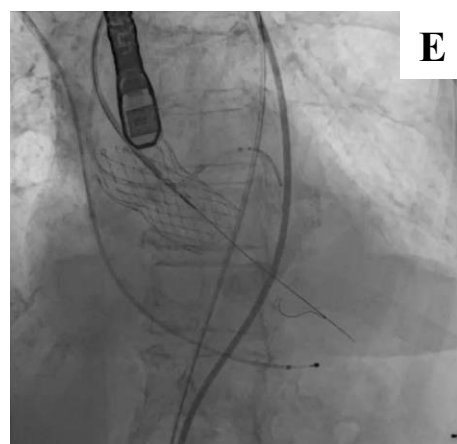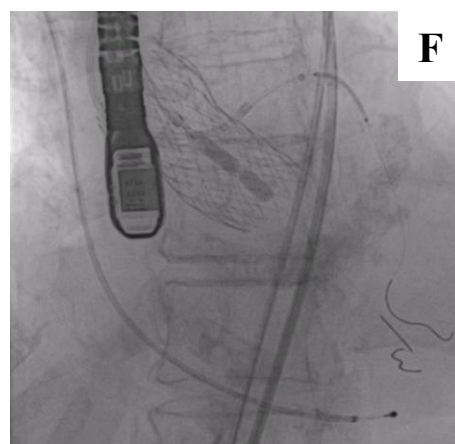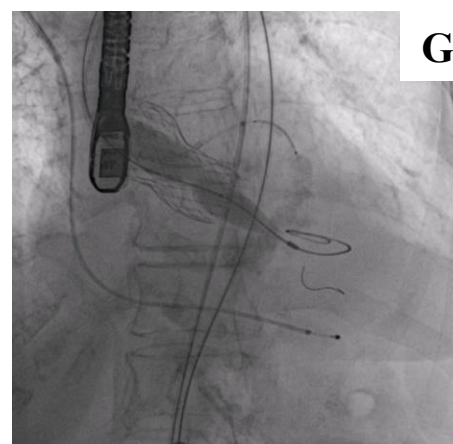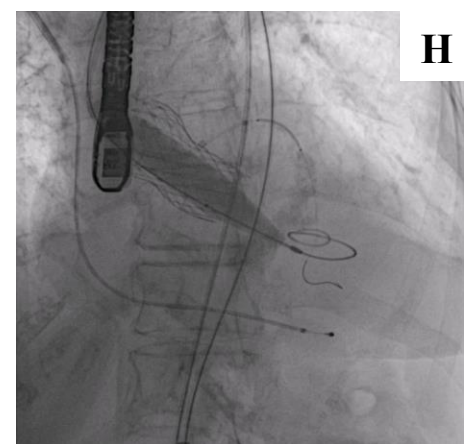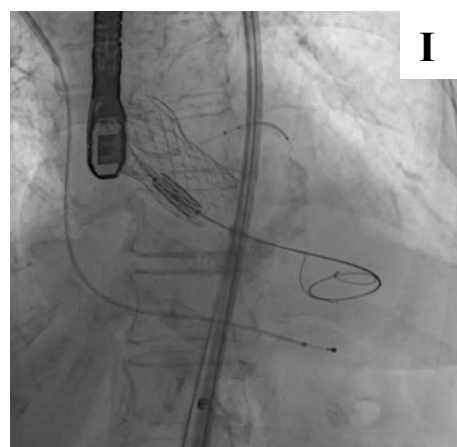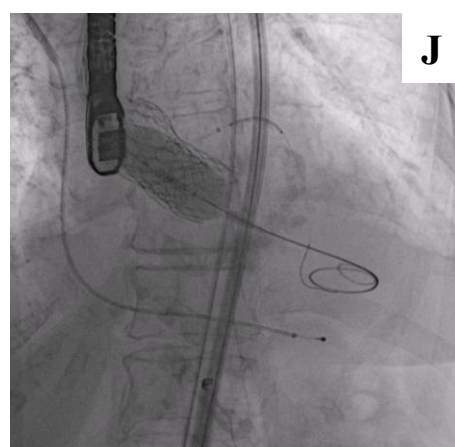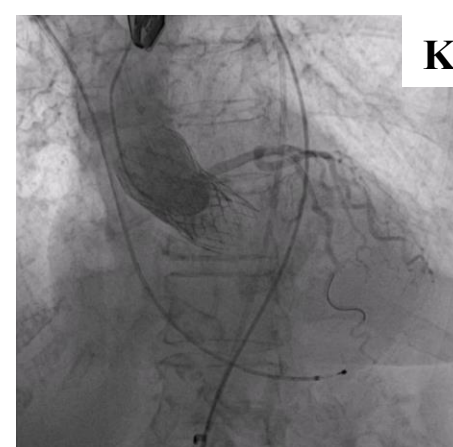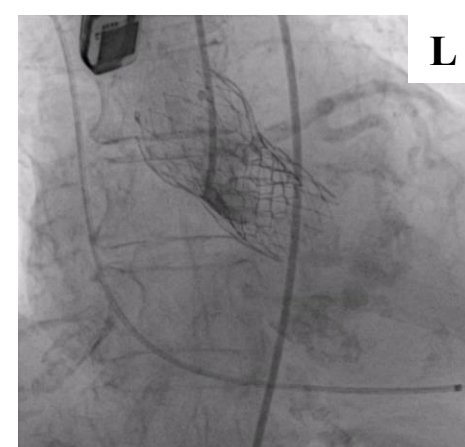

Supplement: Supplemental Figure 1 — Failed UNICORN-assisted TAVR-in-TAVR with post-TAVR coronary obstruction. (a) Nonselective angiogram demonstrating patent right coronary artery. (b) Selective angiogram demonstrating patent left coronary artery. (c) left main protection with GuideLiner catheter and coronary stent (not deployed). (d) Catheter aligned with right coronary cusp. (e) right coronary cusp traversal with Astato-Turnpike using electrosurgery at 50 W. (f) Initial 5.0-mm balloon inflation with laceration of the right coronary cusp. (g, h) right coronary cusp laceration with 16.0-mm balloon. (i, j) Deployment of the transcatheter heart valve. (k) Aortogram with TAVR-in-TAVR and absent right coronary flow. (l) Selective angiogram with TAVR-in-TAVR and absent right coronary flow indicating right coronary obstruction. Abbreviations: TAVR, transcatheter aortic valve replacement; UNICORN, Undermining Iatrogenic Coronary Obstruction with Radiofrequency Needle. [file mmc1.pdf]
